# Ultrasound Applications in Critical Care Medicine

**DOI:** 10.1155/2012/382615

**Published:** 2012-07-12

**Authors:** Richard Hoppmann, Dimitrios Karakitsos

**Affiliations:** ^1^Department of Internal Medicine, School of Medicine, University of South Carolina, Columbia, SC 29208, USA; ^2^Intensive Care Unit, General State Hospital of Athens, 154 Mesogeion Ave., 11427 Athens, Greece

The use of ultrasound has expanded enormously over the last two decades in critical care research and practice. Despite the fact that the method has several inherent limitations and is largely operator dependent, it enables clinicians for rapid, by-the-bed, and relatively inexpensive diagnostic evaluation of unstable patients. Point-of-care ultrasound applications such as lung ultrasound are gradually replacing traditional imaging modalities (i.e., chest X-rays), while the use of ultrasound for procedure guidance has been shown to reduce complications and thus to increase patients' safety [1–3].

In this issue, various papers illustrated the important role of ultrasound in the intensive care unit (ICU). K. Stefanidis et al. applied echogenic technology during ultrasound-guided cannulation of the internal jugular vein (IJV) and of the subclavian vein (SCV), respectively. Both case-control studies included intubated critical care patients and were performed under controlled ICU conditions. Ultrasound-guided cannulation of the IJV was attempted on the transverse axis and cannulation of the SCV on the longitudinal axis via an infraclavicular approach. In both studies, the use of echogenic technology significantly improved cannula visibility and decreased access time and technical complexity optimizing thus real-time ultrasound-guided central venous cannulation irrespective of the technique used.

Current trends promote the optimization of two-dimensional ultrasound imaging by applying various technologies. Advances in ultrasound software can reduce artifacts and “noise” such as speckle arising from coherent wave interference or clutter arising from beamforming artifacts, reverberations, and other acoustic phenomena, while infusion of contrast agents during imaging facilitates interpretation of various pathologies [4, 5]. In that sense, the application of echogenic material could optimize procedural ultrasound applications. This may be of importance as ultrasound scanning is oftentimes performed under suboptimal conditions in the ICU, while the presence of mechanical ventilation, air and/or edema, may affect the clarity of images [6]. The clinical notion that IJV cannulation in patients at risk for intracranial hypertension could impair cerebral venous return was explored by D. Vailati et al. in a prospective study. They used two-dimensional and Doppler techniques to evaluate IJV cross-sectional diameter and flow patterns before and after ultrasound-guided cannulation in neurosurgical patients placed in a supine or a head-up position at 30 degrees. No significant alterations in IJV flow rates following cannulation were recorded; however, the study was focused only on the venous system and no data about the arterial flow were presented. Future studies exploring changes in both vascular circuits are clearly required to clarify further possible changes in cerebral hemodynamics that might be attributed to the cannulation procedure itself and/or to the relevant body position acquired. In an interesting vascular study, M. Blaivas et al. studied retrospectively 320 critical care patients receiving a SCV or IJV central venous catheter (CVC) to evaluate the rate of upper extremity deep venous thrombosis (UEDVT) and the sonographic appearance of thrombi. In this series, 2.7% of patients died and 5.5% had pulmonary embolism, while approximately 11% had UEDVT. Risk factors associated with UEDVT were presence of CVC (odds ratio (OR) 2.716, *P* = 0.007), malignancy (OR 1.483, *P* = 0.036), total parenteral nutrition (OR 1.399, *P* = 0.035), hypercoagulable state (OR 1.284, *P* = 0.045), and obesity (OR 1.191, *P* = 0.049). Eight thrombi were chronic, and 28 were acute. Notably, the authors presented a new sonographic sign which characterized acute thrombosis: a double hyperechoic line at the interface between the thrombus and the venous wall. They concluded that the presence of CVC was a strong predictor for the development of UEDVT, while the actual rate of subsequent PE was low. Surely, contrast venography remains a standard diagnostic technique in the evaluation of UEDVT; however, ultrasound has a clear established diagnostic role, while imaging with gadolinium contrast-enhanced magnetic resonance imaging is routinely used but has not been properly validated yet. Moreover, the recognition of gadolinium as a cause of nephrogenic systemic fibrosis has increased interest in noncontrast magnetic resonance venography [7].

Apart from vascular ultrasound studies discussed above, P. Myrianthefs et al. evaluated whether routine ultrasound examination may illustrate gallbladder abnormalities, including acute acalculous cholecystitis (AAC) in a cohort of critical care patients. The authors evaluated major (gallbladder wall thickening and edema, sonographic Murphy's sign, pericholecystic fluid) and minor (gallbladder distention and sludge) ultrasound criteria. Notably 47.2% of patients showed at least one abnormal imaging finding; however, only 5.7% of cases were identified as AAC. They conclude that diagnosis of AAC requires high levels of clinical suspicion. Nevertheless, this series was small and future larger studies are clearly required to investigate the potential screening role of ultrasound in detecting gallbladder disorders in the ICU.

Reading further on, three interesting papers focus on echocardiography emerge. T. Bagger et al. compared conventional and automated speckle tracking echocardiography to determine whether left ventricular (LV) systolic function could be estimated from one single imaging plane. They found a bias of 0.6 (95% CI −2.2–3.3) for global peak systolic strain comparing the automated and the conventional method. Notably, global peak systolic strain of apical 4-chamber cine-loops versus averaged global peak strain obtained from apical 4, 2, and long axis cine loops showed a bias of 0.1 (95% CI −3.9–4.0), and agreement between 4-chamber subcostal and apical global peak systolic strain was 4.4 (95% CI −3.7–12.5). Hence, they found good agreement between conventional and automated methods; moreover, speckle tracking ultrasound applied to single apical 4-chamber cine loops showed excellent agreement with overall averaged global peak systolic strain. In contrast, subcostal 4-chamber cine loops were rather unsuitable for the automated method. Technical issues related to the subjective evaluation of LV function may be solved by the application of advanced echocardiographic methods. Implementing the latter in routine practice remains debatable but represents an option that should be discussed awaiting the validation of currently applied basic echocardiography training programs for noncardiologists. Despite the fact that only basic elements of echocardiography are currently integrated in point-of-care ultrasound training programs, the method represents a “hot spot” of debate in critical care research and practice. J. C. Mandeville and C. L. Colebourn discussed whether transthoracic echocardiography can predict fluid responsiveness in the critically ill following a thorough literature search. They concluded that inferior vena cava analysis and transaortic Doppler signal changes with the respiratory cycle in mechanically ventilated patients were predictors of fluid responsiveness. Fluid responsiveness in the critically ill is a subject of ongoing research. Oftentimes various pathologies which may affect volume status coexist, while the clinical picture can be easily blurred in ICU patients. Recently, suggestions of noninvasive hemodynamic models comprising of lung and cardiovascular ultrasound emerged [8]. Development of advanced noninvasive hemodynamic monitoring models based on current ultrasound techniques remains to be explored in future studies. Surely, the role of echocardiography in hemodynamic monitoring remains pivotal. Also, clinical entities such as LV diastolic dysfunction become increasingly recognized in ICU patients. In their expert analysis, L. A. Eisen et al. illustrated that heart failure with a normal or nearly normal LV ejection fraction (HFNEF) may represent more than 50% of heart failure cases. However, there is a relative lack of information regarding LV diastolic dysfunction incidence and prognostic implications in critical care patients. In the ICU, many factors related to patient's history, or applied therapies, may induce or aggravate LV diastolic dysfunction, while the latter was linked as well to weaning failure. This may impact on patients' morbidity and mortality. Finally, in this issue, K. Stefanidis et al. evaluated prospectively the utility of lung ultrasound in detecting and localizing alveolar-interstitial syndrome in respective pulmonary lobes as compared to computed tomography scans in ICU patients. The authors designated lobar reflections along intercostal spaces and surface lines by means of sonoanatomy in an effort to accurately localize lung pathology, while the presence of diffuse comet-tail artifacts was considered a sign of alveolar-interstitial syndrome. They found that lung ultrasound showed high sensitivity and specificity values (ranging from over 80% for the lower lung fields up to over 90% for the upper lung fields) and considerable consistency in the diagnosis and localization of alveolar-interstitial syndrome. The diagnostic role of lung ultrasound is well established in the ICU. As the method grows and technology advances, lung ultrasound may represent an alternative to computed tomography in the monitoring of pulmonary disorders, although further studies are clearly required to validate this notion.

Surely, applying point-of-care ultrasound in the ICU requires formal training. Critical care fellowships offered by European and US residency programs are currently taking on the burden of such responsibility [9]. Notably, several US-based institutions are integrating ultrasound teaching programs in medical schools' curricula as this would aid all graduates to obtain basic ultrasound skills [10, 11]. Such skills should not be used as a replacement to standard physical examination and/or to clinical judgment, but as an adjunctive tool that could facilitate patients' diagnosis and treatment. Changing practices by implementing ultrasound technology in the ICU is a cost-efficient and robust strategy which signals an era of pure “visual” medicine.

*Richard Hoppmann* *Dimitrios Karakitsos*
